# Withdrawn: The Association of Gender Based Violence (GBV) with Familial and Social Determinants and Mental Health Status of Bengali Married Working Women: A Cross-sectional Study

**DOI:** 10.2174/0117450179400081250812509051

**Published:** 2025-09-15

**Authors:** Tasnim Akter, Md. Imdadul Haque, Md. Zobaer Hasan, Faisal Muhammad, Sharmin Sultana, Md. Golam Dostogir Harun, Md. Shahinuzzaman, Syeda Humayra, Md. Monirul Islam, Sabina Sharmin, Alauddin Chowdhury ABM

**Affiliations:** 1 Department of Public Health, Faculty of Allied Health Sciences, Daffodil International University (DIU), Dhaka 1207, Bangladesh; 2 School of Science, Monash University Malaysia, Jalan Lagoon Selatan, 47500 Bandar Sunway, Selangor Darul Ehsan, Malaysia; 3 Department of General Educational Development, Daffodil International University, Dhaka, Bangladesh; 4 Department of Psychology, Faculty of Life and Earth Science, Jagannath University, Dhaka 1100, Bangladesh; 5 Department of Sociology, Faculty of Social Sciences, Jagannath University, Dhaka1100, Bangladesh

The article has been withdrawn by the journal **Clinical Practice & Epidemiology in Mental Health**.

The publisher apologizes to the readers of the journal for any inconvenience this may have caused.

The Bentham editorial policy on article withdrawal can be found at https://clinical-practice-and-epidemiology-in-mental-health.com/editorial-policies.php#sec19

**BENTHAM OPEN DISCLAIMER:** It is a condition of publication that manuscripts submitted to this journal have not been previously published and will not be simultaneously submitted or published elsewhere. Furthermore, any data, illustration, structure, or table that has been published elsewhere must be reported, and copyright permission for reproduction must be obtained. Plagiarism is strictly forbidden, and by submitting the article for publication, the authors agree that the publishers have the legal right to take appropriate action against the authors if plagiarism or fabricated information is discovered. By submitting a manuscript, the authors agree that the copyright of their article is transferred to the publishers if and when the article is accepted for publication.

